# The reciprocal longitudinal associations among Exercise Identity, exercise self-efficacy, and physical activity participation in college students: a two-wave cross-lagged panel study

**DOI:** 10.3389/fpsyg.2026.2004472

**Published:** 2026-10-30

**Authors:** Weijie Gao, Caiyan Li, Duo Zhang

**Affiliations:** 1 Chengdu University of Traditional Chinese Medicine, Chengdu, China; 2 Jeonbuk National University, Jeonju, Republic of Korea

**Keywords:** cross-lagged panel model, Exercise Identity, exercise self-efficacy, longitudinal study, physical activity participation

## Abstract

**Purpose:**

This study aimed to examine the temporal reciprocal associations among college students’ Exercise Identity, exercise self-efficacy, and physical activity participation, and to investigate potential psychological pathways underlying these longitudinal associations.

**Methods:**

A two-wave longitudinal design was employed with 475 college students (261 males, 54.95%; 214 females, 45.05%; M_age = 20.33 years, SD = 1.95, range = 18–25 years). Exercise Identity, exercise self-efficacy, and physical activity participation were assessed at two measurement occasions separated by four months. Pearson correlation analyses and a cross-lagged panel model (CLPM) were conducted to examine the temporal reciprocal predictive associations among the three constructs. Bootstrapping procedures were further applied to examine the indirect association of exercise self-efficacy in the relationship between Exercise Identity and physical activity participation.

**Results:**

(1) Exercise Identity, exercise self-efficacy, and physical activity participation were significantly and positively correlated at both measurement occasions (all ps < 0.001). (2) After controlling for the autoregressive effects of each construct, Exercise Identity at T1 significantly predicted exercise self-efficacy at T2 (β = 0.112, *p* = 0.004) and physical activity participation at T2 (β = 0.143, *p* < 0.001). Exercise self-efficacy at T1 significantly predicted subsequent Exercise Identity (β = 0.098, *p* = 0.025) and physical activity participation (β = 0.170, *p* < 0.001). Physical activity participation at T1 also significantly predicted subsequent Exercise Identity (β = 0.096, *p* = 0.021) and exercise self-efficacy (β = 0.172, *p* < 0.001). (3) Exercise self-efficacy showed a significant indirect association between Exercise Identity and physical activity participation, with an indirect effect of 0.037.

**Conclusion:**

Exercise Identity, exercise self-efficacy, and physical activity participation showed reciprocal prospective associations among college students. Exercise Identity was associated with subsequent physical activity participation and showed a significant indirect association through exercise self-efficacy, while physical activity participation was associated with subsequent Exercise Identity and exercise self-efficacy. Given the two-wave observational design and the relatively small cross-lagged effects, these findings should be interpreted as modest prospective associations rather than evidence of causal developmental processes. These findings highlight the importance of considering both identity-related perceptions and competence beliefs when understanding physical activity participation among college students.

## 1 Introduction

### 1.1 Research background

Physical activity, as a fundamental health-promoting behavior, plays a critical role in enhancing physical health and supporting psychological development. Extensive research has demonstrated that regular physical activity not only improves cardiorespiratory fitness and physical functioning but also reduces psychological stress and enhances well-being and quality of life (Warburton and Bredin, 2017). In recent years, as physical inactivity has emerged as a growing global public health concern, promoting stable and sustained engagement in physical activity has become a major research priority in exercise science and public health. The *World Health Organization Guidelines on Physical Activity* emphasize that increasing population-level physical activity requires not only supportive environments for exercise but also attention to the psychological mechanisms underlying behavioral initiation and maintenance (World Health Organization, 2020).

College students are in a critical period of physical development and social transition, making this stage particularly important for cultivating lifelong physical activity awareness and establishing exercise habits. However, existing research indicates that although college students generally recognize the importance of physical activity, their actual participation levels remain inadequate. Upon entering college, increased academic demands, altered daily routines, and reduced external supervision may lead some students to gradually decrease their physical activity frequency and experience difficulties maintaining regular exercise habits (Dawson et al., 2023). This phenomenon suggests that physical education courses, sports facilities, and external management strategies alone cannot fully explain individual differences in the maintenance of physical activity participation. Therefore, investigating the psychological mechanisms underlying the formation and maintenance of physical activity behavior is essential for understanding and promoting long-term physical activity participation among college students.

Traditional research in exercise psychology has primarily explained physical activity participation through constructs such as exercise motivation, self-efficacy, and behavioral intention. Among these factors, exercise self-efficacy has been identified as a key psychological determinant of physical activity behavior (Dai et al., 2025). Within the context of physical activity, individuals with higher exercise self-efficacy generally demonstrate greater confidence in overcoming barriers such as time constraints, fatigue, and environmental limitations, thereby increasing their likelihood of maintaining regular physical activity participation. Recent studies among college students have further demonstrated that exercise self-efficacy plays an important role in the association between physical activity and positive psychological outcomes, suggesting that self-efficacy may represent a key mechanism linking physical activity behavior with psychological development (Yu et al., 2024).

However, an ability-based perspective, which focuses solely on “believing one can exercise,” may be insufficient to explain why some individuals successfully integrate physical activity into their daily lives over the long term. In recent years, exercise psychology research has increasingly emphasized more stable identity-based factors, particularly exercise identity. Exercise identity reflects the extent to which individuals perceive being an “exerciser” as an integral component of their self-concept. When individuals develop a strong exercise identity, physical activity may shift from a behavior driven primarily by external demands or short-term interests to a behavior that supports self-consistency and identity expression.

In recent years, increasing attention has been directed toward the dynamic associations between Exercise Identity and physical activity behavior. A meta-analysis by Rhodes et al. (2016) demonstrated a consistent association between Exercise Identity and physical activity behavior, identifying Exercise Identity as an important psychological correlate of physical activity levels. Furthermore, Porter et al. (2024) employed a four-wave longitudinal cross-lagged panel model to examine the reciprocal temporal associations between Exercise Identity and physical activity behavior. They found that Exercise Identity significantly predicted subsequent physical activity behavior, whereas physical activity behavior showed a relatively weaker predictive association with subsequent Exercise Identity, suggesting that identity-related factors may contribute importantly to the maintenance of exercise behavior.

Although previous research has substantially advanced understanding of the relationship between Exercise Identity and physical activity participation, two important gaps remain. First, Exercise Identity and exercise self-efficacy have often been examined separately, leaving their potentially complementary roles within the same longitudinal framework insufficiently understood. In particular, it remains unclear whether exercise self-efficacy may represent a potential explanatory pathway linking earlier Exercise Identity with later physical activity participation. Second, much of the existing evidence is cross-sectional, which limits examination of temporal ordering and prospective reciprocal associations among Exercise Identity, exercise self-efficacy, and physical activity behavior. Longitudinal evidence addressing these relationships remains especially limited among college students.

Therefore, the present study integrates Exercise Identity, exercise self-efficacy, and physical activity participation within a unified longitudinal framework. It extends previous research by examining identity-based and competence-based processes jointly, testing prospective reciprocal associations among the three constructs, and exploring exercise self-efficacy as a potential explanatory pathway linking earlier Exercise Identity with later physical activity participation. Using a two-wave cross-lagged panel design, the study aims to clarify the temporal pattern of these associations among college students. Given the observational nature of the design, the findings are interpreted as prospective longitudinal associations rather than causal effects.

### 1.2 Theoretical foundations and mechanisms of exercise identity

The theoretical foundations of Exercise Identity are primarily derived from Identity Theory and Social Identity Theory. Identity Theory proposes that individuals possess multiple identities, and the salience of these identities within the self-concept varies across individuals and contexts (Demir Kaya and Kaya, 2026). When a particular identity becomes integrated into an individual’s core self-concept, it can exert a persistent influence on behavior by motivating individuals to adopt actions consistent with that identity (Stryker and Burke, 2000).

Within the exercise context, a salient Exercise Identity may therefore provide a self-definitional basis for identity-consistent physical activity behavior. Individuals with a stronger Exercise Identity are more likely to regard exercise as an important expression of the self and to maintain behaviors consistent with that identity (Mansfield et al., 2022). This identity-based mechanism helps explain why some individuals maintain long-term physical activity participation even in the absence of external regulation or supervision. Because physical activity often involves social interaction, participation in sports clubs, team-based activities, and campus athletic programs may provide college students with peer support and group recognition, thereby further strengthening their Exercise Identity.

Taken together, Identity Theory and Social Identity Theory suggest that Exercise Identity may be shaped by both personal self-definition and social identification. Importantly, the association between Exercise Identity and physical activity may also be reciprocal: a stronger Exercise Identity may be prospectively associated with greater subsequent participation, whereas repeated physical activity experiences may, in turn, be associated with the subsequent strengthening of Exercise Identity. Thus, Exercise Identity is better conceptualized as part of a dynamic longitudinal relationship with physical activity rather than solely as a unidirectional predictor of behavior.

### 1.3 Research progress on the relationship among exercise self-efficacy, exercise Identity, and physical activity behavior

Exercise self-efficacy represents an important psychological construct for explaining variations in physical activity behavior. Social cognitive theory proposes that self-efficacy influences not only behavioral choices but also individuals’ persistence and effort when encountering challenges (Bandura, 1997). Within the context of physical activity, individuals with higher exercise self-efficacy generally demonstrate greater confidence in overcoming barriers and stronger persistence in maintaining exercise behaviors.

In recent years, researchers have increasingly recognized that exercise self-efficacy and Exercise Identity may jointly contribute to the development and maintenance of physical activity behavior. Exercise Identity reflects individuals’ perceptions of “who I am,” whereas exercise self-efficacy reflects beliefs regarding “whether I can do it”; these constructs represent identity-based and competence-based psychological mechanisms, respectively (Woo et al., 2024). Theoretically, a stronger Exercise Identity may enhance individuals’ perceived value of physical activity, thereby strengthening confidence in their exercise capabilities and increasing exercise self-efficacy; conversely, repeated successful exercise experiences may enhance perceived competence and further reinforce Exercise Identity development. Therefore, Exercise Identity and exercise self-efficacy may jointly contribute to physical activity participation through complementary psychological pathways (Lochbaum et al., 2023).

Recent empirical studies have provided preliminary support for this theoretical perspective. Li et al. (2024), examining exercise persistence among college students, found that exercise self-efficacy significantly contributed to the association between exercise engagement and exercise persistence, highlighting the importance of psychological beliefs in maintaining physical activity participation. Furthermore, Gao et al. (2025), using a longitudinal cross-lagged design, identified reciprocal associations among physical activity, self-efficacy, and psychological outcomes, further suggesting that the interplay between psychological factors physical activity is dynamic rather than unidirectional.

Nevertheless, relatively few studies have simultaneously examined Exercise Identity, exercise self-efficacy, and physical activity participation within the same longitudinal framework. Consequently, the temporal relationships among identity-based self-definition, competence-based beliefs, and physical activity behavior remain insufficiently understood. Examining these constructs together may therefore provide a more integrated account of the psychological processes associated with physical activity participation over time.

### 1.4 Rationale for using the cross-lagged panel model

Traditional cross-sectional studies can identify associations among variables but provide limited information about their prospective temporal relationships or potential reciprocal patterns. In sport psychology, psychological and behavioral factors may be related to one another over time; therefore, single-time-point assessments are insufficient for examining how earlier levels of one construct are associated with later levels of another.

The cross-lagged panel model (CLPM) is a longitudinal analytical approach that examines temporal predictive associations among variables across multiple measurement occasions while controlling for the autoregressive effects of each construct (Hamaker et al., 2015). By accounting for the temporal stability of the constructs, CLPM allows the prospective association of one variable with another at a later measurement occasion to be evaluated. However, particularly in a traditional two-wave design, these cross-lagged associations should not be interpreted as evidence of causal effects.

In the present study, CLPM provides a framework for examining whether earlier Exercise Identity, exercise self-efficacy, and physical activity participation are associated with subsequent levels of one another after accounting for their baseline stability. It therefore enables the examination of reciprocal cross-lagged patterns among identity-based perceptions, competence beliefs, and physical activity behavior. The indirect association involving exercise self-efficacy is examined separately as a potential explanatory pathway rather than as evidence of a fully temporally ordered causal mechanism.

### 1.5 Research objectives and hypotheses

This study adopts a two-wave longitudinal design and employs a cross-lagged panel model (CLPM) to examine the reciprocal longitudinal associations among college students’ Exercise Identity, exercise self-efficacy, and physical activity participation. By examining cross-lagged predictive pathways while controlling for the temporal stability of each construct, this study aims to provide insights into the temporal ordering of relationships among these psychological and behavioral factors.

Based on the theoretical framework and previous empirical findings, the following hypotheses are proposed:

H1: Exercise Identity, exercise self-efficacy, and physical activity participation are expected to be significantly and positively associated among college students.

H2: After controlling for baseline levels of physical activity participation, Exercise Identity at T1 will positively predict physical activity participation at T2.

H3: After controlling for baseline levels of Exercise Identity, physical activity participation at T1 will positively predict Exercise Identity at T2.

H4: Exercise Identity at T1 will positively predict exercise self-efficacy at T2 after controlling for baseline levels of exercise self-efficacy.

H5: After controlling for T1 exercise self-efficacy and T1 physical activity participation, Exercise Identity at T1 is expected to show a positive indirect association with physical activity participation at T2 via exercise self-efficacy measured at T2.

## 2 Research methods

### 2.1 Research participants

This study employed a two-wave longitudinal design involving undergraduate students from multiple universities as participants. The college years represent a critical developmental period for establishing physical activity habits and developing Exercise Identity; therefore, college students were selected as the target population due to their theoretical and practical relevance to the research objectives. Data were collected at two measurement occasions (T1 and T2), separated by an interval of approximately 4 months. At T1, participants provided demographic information and completed measures of Exercise Identity, exercise self-efficacy, and physical activity participation. The same measures were administered again at T2, and longitudinal data were matched using individual identification codes.

The inclusion criteria were as follows: (1) being a full-time undergraduate student aged 18–25 years; (2) having no major physical conditions that could interfere with regular physical activity participation; and (3) being able to complete both questionnaire assessments. The exclusion criteria were as follows: (1) extremely short questionnaire completion times; (2) evidence of patterned responding; and (3) missing data on key variables. A total of 500 participants were recruited and completed the baseline assessment at Time 1 (T1). Four months later, participants were invited to complete the follow-up assessment at Time 2 (T2). A total of 475 participants completed both assessments, whereas 25 participants were lost to follow-up, resulting in an attrition rate of 5.0%. The final analytical sample consisted of 475 participants, including 261 males (54.95%) and 214 females (45.05%). Participants had a mean age of 20.33 years (SD = 1.95; range = 18–25 years). Regarding year of study, 144 participants (30.32%) were first-year students, 156 (32.84%) were second-year students, 97 (20.42%) were third-year students, and 78 (16.42%) were fourth-year students. Participants represented a range of academic disciplines, including education, psychology, engineering, mathematics, and arts, among others.

An *a priori* power analysis was conducted using G*Power 3.1 based on multiple regression assumptions. Assuming a medium effect size (f^2^ = 0.15), an alpha level of 0.05, and statistical power of 0.80, the minimum required sample size was approximately 68 participants. The final sample size (*N* = 475) exceeded this requirement, providing sufficient power for the planned analyses.

Participants were recruited using both online and offline recruitment procedures. Before data collection, participants were informed of the study objectives, procedures, and confidentiality protections. All participants voluntarily participated and provided written informed consent. The study protocol was approved by the institutional ethics committee (Approval No. KIRD-2024-03-31-J-E-346).

### 2.2 Research design and procedure

This study employed a two-wave longitudinal design to examine the dynamic reciprocal relationships among college students’ Exercise Identity, exercise self-efficacy, and physical activity participation over time. Unlike cross-sectional designs, which capture associations at a single time point, longitudinal designs enable the examination of changes and relationships across multiple measurement occasions. By controlling for the autoregressive effects of variables, longitudinal models allow researchers to examine prospective cross-lagged associations between psychological and behavioral factors over time. Accordingly, this study employed a cross-lagged panel model (CLPM) to examine the reciprocal predictive associations among Exercise Identity, exercise self-efficacy, and physical activity participation.

The research procedure consisted of the following stages:

In Phase 1 (T1), data collection was conducted using an online survey platform. Participants first provided demographic information, including gender, age, academic year, and major, and subsequently completed measures of Exercise Identity, exercise self-efficacy, and physical activity participation. Data collected at T1 were used to establish baseline levels and served as predictors in subsequent longitudinal analyses.

Four months later, a follow-up assessment was conducted at T2. After matching participants across waves, the same measures were administered again to construct a two-wave longitudinal dataset. The 4-month interval was selected based on theoretical and methodological considerations. This interval was selected because Exercise Identity and exercise self-efficacy are relatively stable psychological constructs that may require time to change, whereas physical activity participation may vary with changes in daily routines and academic contexts. A 4-month interval, corresponding approximately to one academic semester, was considered appropriate for examining prospective associations among these constructs and was consistent with previous longitudinal research (Porter et al., 2024).

During data analysis, a cross-lagged panel model was used to incorporate T1 measurements of the three core variables and examine their prospective associations with T2 measures while controlling for baseline levels of each construct. This approach controls for the temporal stability of each construct and evaluates cross-lagged predictive pathways among the variables. Specifically, the model examined the following longitudinal associations:

(1) Whether Exercise Identity at T1 predicts subsequent physical activity participation at T2;
(2) Whether physical activity participation at T1 predicts subsequent Exercise Identity at T2, indicating whether behavioral experiences are associated with identity development;
(3) Whether Exercise Identity at T1 predicts subsequent exercise self-efficacy at T2, indicating whether identity-related perceptions are associated with changes in competence beliefs;
(4) Whether exercise self-efficacy represents a potential indirect pathway linking Exercise Identity at T1 with physical activity participation at T2.

Through this design, the study aims to address the limitations of cross-sectional research in examining temporal directionality and to elucidate the dynamic psychological mechanisms underlying the development and maintenance of physical activity participation among college students. This design corresponds with hypotheses H2–H5, which examine prospective longitudinal associations among Exercise Identity, exercise self-efficacy, and physical activity participation.

### 2.3 Measurement instruments

#### 2.3.1 Exercise identity scale

This study used the Exercise Identity Scale (EIS) developed by Anderson and Cychosz (1994) to assess college students’ exercise identity. The Exercise Identity Scale is one of the most widely used instruments in exercise psychology and assesses the extent to which individuals integrate exercise into their self-concept. The scale is grounded in identity theory, which proposes that identification with the exerciser role shapes individuals’ behavioral choices and the maintenance of physical activity behaviors. The Chinese version was validated among Chinese college students by Li et al. (2019). Their psychometric evaluation supported a unidimensional structure and demonstrated satisfactory internal consistency, test–retest reliability, and construct validity.

The scale consists of 9 items rated on a 7-point Likert scale ranging from 1 (“strongly disagree”) to 7 (“strongly agree”), with higher scores indicating a stronger Exercise Identity. In the present sample, internal consistency was good at both waves, with Cronbach’s α = 0.887 and McDonald’s ω = 0.888 at T1, and Cronbach’s α = 0.890 and McDonald’s ω = 0.890 at T2. Separate one-factor CFAs also supported the factorial validity of the scale at T1 (CFI = 0.977, TLI = 0.970, RMSEA = 0.055, SRMR = 0.027) and T2 (CFI = 0.971, TLI = 0.961, RMSEA = 0.064, SRMR = 0.029). The items primarily assess individuals’ perceptions of themselves as exercisers, the personal significance of exercise, and the role of exercise identity within their self-concept.

Previous research has demonstrated that the Exercise Identity Scale exhibits satisfactory internal consistency and construct validity and has been widely applied in studies of physical activity behavior across diverse age groups (Anderson and Cychosz, 1994). Recent longitudinal research has further supported the predictive association between exercise identity and long-term physical activity behavior, suggesting that the scale effectively captures stable individual differences in exercise identity (Porter et al., 2024).

#### 2.3.2 Exercise self-efficacy scale

This study used the Exercise Self-Efficacy Scale developed by Marcus et al. (1992) to assess college students’ confidence in maintaining physical activity across various situations. Exercise self-efficacy reflects individuals’ beliefs regarding their capability to initiate and maintain exercise behaviors and represents a key psychological construct in social cognitive theory’s explanation of physical activity behavior. The Chinese version consists of 8 items and assesses individuals’ perceived confidence in engaging in physical activity across common situations and barriers. Chen et al. (2017) supported the unidimensional structure and measurement properties of the Chinese version across gender and educational levels.

The scale primarily assesses individuals’ confidence in maintaining physical activity when encountering common exercise barriers, such as time constraints, academic stress, fatigue, and limited access to exercise opportunities. Participants rate their confidence in maintaining exercise under different circumstances using a Likert-type scale, with higher scores indicating greater exercise self-efficacy. In the present sample, Cronbach’s α and McDonald’s ω were 0.877 and 0.878 at T1, respectively, and 0.893 and 0.893 at T2. Separate one-factor CFAs indicated satisfactory factorial validity at T1 (CFI = 0.973, TLI = 0.963, RMSEA = 0.065, SRMR = 0.028) and T2 (CFI = 0.988, TLI = 0.983, RMSEA = 0.047, SRMR = 0.023).

#### 2.3.3 Measurement of physical activity participation

Physical activity participation was assessed using the Chinese version of the International Physical Activity Questionnaire–Short Form (IPAQ-SF). The original IPAQ was developed and internationally validated by Craig et al. (2003), while the Chinese short-form version was developed and psychometrically evaluated by Macfarlane et al. (2007). Their validation study provided evidence of acceptable reliability and validity for estimating total physical activity in Chinese populations.

The IPAQ-SF assesses participation in physical activities of different intensities during the previous 7 days, including vigorous-intensity activity, moderate-intensity activity, walking, and sedentary behavior. Specifically, participants report the frequency (days per week) and duration (minutes per day) of each type of physical activity. According to IPAQ-SF scoring guidelines, metabolic equivalent of task (MET) values were assigned to activities of different intensities, with vigorous-intensity activity, moderate-intensity activity, and walking assigned values of 8.0, 4.0, and 3.3 METs, respectively. Because MET-min/week values typically show a highly skewed distribution, the physical activity participation index was log-transformed using ln(1 + MET-min/week) before subsequent analyses.

#### 2.3.4 Demographic variables

In addition to the primary study variables, demographic information, including gender, age, academic year, and major, was collected from participants. Previous research suggests that demographic characteristics may be associated with college students’ physical activity participation and exercise-related psychological factors; therefore, these variables were included as covariates in the analytical models to account for potential confounding effects.

### 2.4 Statistical analysis

Statistical analyses were conducted using SPSS 27.0 and Mplus 8.3. Descriptive statistics, Pearson correlations, reliability analyses, and attrition analyses were performed using SPSS. Longitudinal measurement invariance of the Exercise Identity and Exercise Self-Efficacy scales was examined across T1 and T2 by sequentially testing configural, metric, scalar, and strict invariance models using robust maximum likelihood estimation (MLR). Changes in CFI and RMSEA, together with robust chi-square difference tests, were used to evaluate invariance across nested models.

A cross-lagged panel model (CLPM) was conducted using structural equation modeling in Mplus to examine the prospective reciprocal associations among Exercise Identity, exercise self-efficacy, and physical activity participation. The model included autoregressive paths to control for the stability of each construct across time. Because the CLPM was fully saturated, global model fit indices were not interpreted; model evaluation focused on standardized autoregressive and cross-lagged path coefficients. The indirect association involving exercise self-efficacy was tested using a bias-corrected bootstrap procedure with 5,000 resamples. No additional covariates were included in the model.

## 3 Research findings

### 3.1 Sample tracking and dropout analysis

To examine whether attrition during longitudinal follow-up introduced potential systematic bias, differences in key study variables at T1 were compared between participants who completed the T2 assessment and those who did not complete the follow-up assessment. As shown in Table 1, 475 participants completed both measurement waves, whereas 25 participants were lost to follow-up. Independent-samples *t*-tests indicated no significant differences at T1 between the two groups in Exercise Identity, exercise self-efficacy, or physical activity participation (all ps = 0.143–0.406). Furthermore, effect sizes were small across all variables (Cohen’s d = 0.167–0.289), suggesting minimal differences in baseline characteristics between participants retained and those lost to follow-up. These findings suggest that attrition was unlikely to have resulted in substantial selection bias, and subsequent analyses were conducted using the 475 participants who completed both measurement waves.

**TABLE 1:** Baseline comparisons between participants retained and lost to follow-up.

Variable	Retention group (n = 475)	Attrition group (n = 25)	*t*	*p*	Cohen’s *d*
T1 Exercise Identity	4.445 ± 1.187	4.787 ± 1.099	−1.507	0.143	−0.289
T1 Exercise self-efficacy	3.206 ± 0.919	3.430 ± 0.984	−1.112	0.276	−0.243
T1 ln(1 + MET-min/week)	7.319 ± 0.588	7.204 ± 0.668	0.844	0.406	0.194
T1 MET-min/week	1758.218 ± 929.778	1603.560 ± 882.172	0.852	0.402	0.167

Physical activity is modeled as ln(1+MET) in the cross-lagged model.

### 3.2 Common method bias and measurement quality

#### 3.2.1 Common method bias test

To assess the potential influence of common method bias, Harman’s single-factor test was conducted as a preliminary assessment. Results indicated that, based on unrotated principal component analysis, the first factor accounted for 36.07% of the total variance at T1 and 37.14% at T2, both below the commonly used 40% threshold. Therefore, no single factor accounted for the majority of the variance, suggesting that common method bias was unlikely to substantially influence the study findings.

#### 3.2.2 Reliability and validity tests of measurement instruments

To evaluate the psychometric properties of the measurement instruments, reliability and validity analyses were conducted for the Exercise Identity Scale and Exercise Self-Efficacy Scale. Internal consistency was assessed using Cronbach’s α and McDonald’s ω coefficients at both T1 and T2.

The Cronbach’s α coefficients for the Exercise Identity Scale were 0.887 at T1 and 0.890 at T2, while those for the Exercise Self-Efficacy Scale were 0.877 and 0.893, respectively. All reliability coefficients exceeded the recommended criterion of 0.70, indicating satisfactory internal consistency.

Exploratory factor analysis showed that the Kaiser–Meyer–Olkin (KMO) values were 0.929 at T1 and 0.933 at T2, and Bartlett’s test of sphericity was significant at both time points (ps < 0.001). A two-factor structure was identified at both measurement occasions, corresponding to Exercise Identity and exercise self-efficacy.

Confirmatory factor analysis was conducted to further examine construct validity. The two-factor measurement model demonstrated good fit at both time points. At T1, the model fit indices were x^2^/df = 1.786, RMSEA = 0.040, CFI = 0.973, TLI = 0.969, and SRMR = 0.039. At T2, the model fit indices were x^2^/df = 1.713, RMSEA = 0.039, CFI = 0.976, TLI = 0.973, and SRMR = 0.040. These results supported the construct validity of the measurement model and its suitability for subsequent analyses.

#### 3.2.3 Longitudinal measurement invariance

Longitudinal measurement invariance was examined for the Exercise Identity Scale and Exercise Self-Efficacy Scale across T1 and T2. Configural, metric, scalar, and strict invariance models were tested sequentially using robust maximum likelihood estimation. As shown in Tables 2 and 3, all models demonstrated satisfactory fit, and changes in model fit across successive levels of invariance were minimal. The robust chi-square difference tests for all nested model comparisons were nonsignificant (ps = 0.136–0.818), while changes in CFI and RMSEA were also very small. These results supported strict longitudinal measurement invariance for both scales, indicating that Exercise Identity and exercise self-efficacy were measured comparably across the two measurement occasions.

**TABLE 2:** Fit indices for longitudinal measurement invariance models.

Scale	Model	Robust x^2^	df	CFI	TLI	RMSEA	SRMR
Exercise Identity	Configural	232.072	125	0.97	0.963	0.043	0.031
Exercise Identity	Metric	242.247	133	0.969	0.965	0.042	0.036
Exercise Identity	Scalar	251.2	141	0.969	0.966	0.041	0.036
Exercise Identity	Strict	257.886	150	0.97	0.969	0.039	0.037
Exercise Self-Efficacy	Configural	148.021	95	0.983	0.979	0.035	0.029
Exercise Self-Efficacy	Metric	156.674	102	0.983	0.98	0.034	0.034
Exercise Self-Efficacy	Scalar	166.004	109	0.982	0.981	0.034	0.035
Exercise Self-Efficacy	Strict	178.329	117	0.981	0.981	0.034	0.038

Configural, metric, scalar, and strict invariance models were tested sequentially. Corresponding item residuals were allowed to correlate across measurement occasions.

**TABLE 3:** Robust difference tests between successive longitudinal measurement invariance models.

Scale	Comparison	Robust Δ x^2^	Δ df	*p*	Δ CFI	Δ RMSEA
Exercise Identity	Metric vs. Configural	9.419	8	0.308	−0.0003	−0.0011
Exercise Identity	Scalar vs. Metric	8.865	8	0.354	−0.0002	−0.0010
Exercise Identity	Strict vs. Scalar	5.186	9	0.818	0.0009	−0.0018
Exercise Self-Efficacy	Metric vs. Configural	7.938	7	0.338	−0.0002	−0.0010
Exercise Self-Efficacy	Scalar vs. Metric	9.281	7	0.233	−0.0007	−0.0005
Exercise Self-Efficacy	Strict vs. Scalar	12.351	8	0.136	−0.0011	−0.0002

Robust Δx^2^ values were obtained using the Satorra–Bentler scaled chi-square difference test for MLR-estimated nested models. ΔCFI and ΔRMSEA were calculated relative to the preceding, less constrained model.

Because physical activity participation was operationalized using the IPAQ-derived MET-min/week score rather than a reflective multi-item latent scale, longitudinal measurement invariance testing was not applicable. The same IPAQ scoring procedure and MET weights were used at both T1 and T2.

### 3.3 Descriptive statistics and changes across the two waves

Table 4 presents the descriptive statistics of the primary study variables across the two measurement waves. At T1, the mean scores for Exercise Identity, exercise self-efficacy, and physical activity participation (MET-min/week) were 4.462, 3.217, and 1,750.485, respectively; at T2, the corresponding means were 4.577, 3.326, and 1,850.304. The results indicated that Exercise Identity, exercise self-efficacy, and physical activity participation showed increasing trends across the two measurement waves.

**TABLE 4:** Descriptive statistics of the main study variables.

Variable	N	M	SD	Min	Max	Skewness	Kurtosis
T1 Exercise Identity	500	4.462	1.184	1.222	7.000	−0.286	−0.467
T1 Exercise self-efficacy	500	3.217	0.922	1.125	5.000	−0.219	−0.635
T1 Physical activity participation (MET-min/week)	500	1750.485	927.224	113.000	5086	0.791	0.264
T1 ln(1 + MET-min/week)	500	7.314	0.592	4.736	8.534	−0.681	0.760
T2 Exercise Identity	475	4.577	1.198	1.111	7.000	−0.197	−0.543
T2 Exercise self-efficacy	475	3.326	0.974	1.000	5.000	−0.161	−0.865
T2 Physical activity participation (MET-min/week)	475	1850.304	1058.974	80.000	6112.5	0.995	1.335
T2 ln(1 + MET-min/week)	475	7.340	0.657	4.394	8.718	−0.849	1.390

Furthermore, distributional analyses indicated that skewness and kurtosis values for Exercise Identity and exercise self-efficacy were within acceptable ranges at both waves, whereas the untransformed physical activity indicator (MET-min/week) showed moderate positive skewness. Following log transformation, the skewness and kurtosis values of the physical activity participation indicator, ln(1 + MET-min/week), improved and met the assumptions required for subsequent parametric analyses; therefore, ln(1 + MET-min/week) was used as the physical activity participation indicator in the cross-lagged panel model analyses.

Table 5 further presents changes in the primary study variables across the two measurement waves among participants who completed both assessments (n = 475). Results of paired-samples t-tests indicated that Exercise Identity significantly increased from 4.445 ± 1.187 at T1 to 4.577 ± 1.198 at T2 (*t* = 2.473, *p* = 0.014); exercise self-efficacy increased from 3.206 ± 0.919 to 3.326 ± 0.974, also demonstrating a significant increase (*t* = 2.931, *p* = 0.004). In contrast, physical activity participation (ln[MET-min/week]) did not differ significantly between the two measurement waves (t = 0.695, *p* = 0.487). Overall, during the 4-month follow-up period, college students demonstrated slight increases in Exercise Identity and exercise self-efficacy, whereas physical activity participation remained stable.

**TABLE 5:** Changes in study variables across two measurement waves among participants completing both assessments (*n* = 475).

Variable	T1	T2	Mean difference	*t*	*p*	Cohen’s *d*z
Exercise Identity	4.445 ± 1.187	4.577 ± 1.198	0.132	2.473	0.014	0.113
Exercise self-efficacy	3.206 ± 0.919	3.326 ± 0.974	0.12	2.931	0.004	0.134
ln(1 + MET-min/week)	7.319 ± 0.588	7.340 ± 0.657	0.021	0.695	0.487	0.032

### 3.4 Correlation analysis

Table 6 presents the Pearson correlation coefficients among the primary study variables across the two measurement waves. Overall, Exercise Identity, exercise self-efficacy, and physical activity participation were significantly and positively correlated at both measurement waves.

**TABLE 6:** Pearson correlation matrix of study variables across two measurement waves (n = 475).

Variable	1	2	3	4	5	6
1. T1 Exercise Identity	–	–	–	–	–	–
2. T1 Exercise self-efficacy	0.352***					
3. T1 Physical activity participation	0.254***	0.329***				
4. T2 Exercise Identity	0.525***	0.294***	0.246***			
5. T2 Exercise self-efficacy	0.318***	0.559***	0.352***	0.345***		
6. T2 Physical activity participation	0.294***	0.339***	0.452***	0.349***	0.480***	

Physical activity participation was measured using ln(1 + MET-min/week). *p* < 0.05, ***p < 0.01, *p* < 0.001.

At T1, Exercise Identity was significantly and positively correlated with both exercise self-efficacy (r = 0.352, *p* < 0.001) and physical activity participation (r = 0.254, *p* < 0.001); exercise self-efficacy was also significantly and positively correlated with physical activity participation (r = 0.329, *p* < 0.001). At T2, the three variables demonstrated similar significant positive associations, with correlations of 0.345 between Exercise Identity and exercise self-efficacy, 0.349 between Exercise Identity and physical activity participation, and 0.480 between exercise self-efficacy and physical activity participation (all ps < 0.001).

Cross-temporal correlation analyses further indicated that all three study variables demonstrated significant temporal stability. Specifically, Exercise Identity at T1 was significantly correlated with Exercise Identity at T2 (r = 0.525, *p* < 0.001); exercise self-efficacy at T1 was significantly correlated with exercise self-efficacy at T2 (r = 0.559, *p* < 0.001); and physical activity participation at T1 was significantly correlated with physical activity participation at T2 (r = 0.452, *p* < 0.001). These findings indicate that Exercise Identity, exercise self-efficacy, and physical activity participation demonstrate both concurrent associations and temporal stability, providing empirical support for subsequent cross-lagged panel model analyses.

### 3.5 Cross-lagged panel model analysis

A two-wave cross-lagged panel model (CLPM) was constructed to examine the prospective reciprocal associations among Exercise Identity, exercise self-efficacy, and physical activity participation. The model included T1 measurements as predictors of corresponding T2 measurements while controlling for autoregressive paths. The variance inflation factors (VIFs) ranged from 1.149 to 1.228, indicating that multicollinearity was not a concern.

Because the CLPM was fully saturated, global model fit indices were not interpreted. Model evaluation focused on standardized path coefficients (β), autoregressive paths, and cross-lagged associations. As shown in Table 7, all autoregressive paths were significant, indicating moderate stability of the three constructs across the 4-month interval.

**TABLE 7:** Standardized path coefficients of the two-wave cross-lagged panel model.

Outcome variable	Predictor variable	Path type	β	SE	*z*	*p*	R^2^
T2 Exercise Identity	T1 Exercise Identity	Autoregressive path	0.466	0.038	12.339	<0.001	0.297
T2 Exercise Identity	T1 Exercise self-efficacy	Cross-lagged path	0.098	0.044	2.238	0.025	0.297
T2 Exercise Identity	T1 Physical activity participation	Cross-lagged path	0.096	0.041	2.31	0.021	0.297
T2 Exercise self-efficacy	T1 Exercise Identity	Cross-lagged path	0.112	0.039	2.887	0.004	0.355
T2 Exercise self-efficacy	T1 Exercise self-efficacy	Autoregressive path	0.463	0.037	12.476	<0.001	0.355
T2 Exercise self-efficacy	T1 Physical activity participation	Cross-lagged path	0.172	0.041	4.189	<0.001	0.355
T2 Physical activity participation	T1 Exercise Identity	Cross-lagged path	0.143	0.04	3.575	<0.001	0.262
T2 Physical activity participation	T1 Exercise self-efficacy	Cross-lagged path	0.17	0.045	3.788	<0.001	0.262
T2 Physical activity participation	T1 Physical activity participation	Autoregressive path	0.36	0.043	8.445	<0.001	0.262

β represents standardized path coefficients. The same R^2^ value is repeated for each predictor path corresponding to the same endogenous variable.

As shown in Table 7, all three study variables demonstrated significant temporal stability across the two measurement waves. Specifically, Exercise Identity at T1 significantly predicted Exercise Identity at T2 (β = 0.466, *p* < 0.001), exercise self-efficacy at T1 significantly predicted exercise self-efficacy at T2 (β = 0.463, *p* < 0.001), and physical activity participation at T1 significantly predicted physical activity participation at T2 (β = 0.360, *p* < 0.001). These findings indicate that Exercise Identity, exercise self-efficacy, and physical activity participation showed substantial temporal stability across measurement waves.

After controlling for the baseline levels of each construct, all six cross-lagged paths were statistically significant (see Figure 1). Specifically, Exercise Identity at T1 positively predicted exercise self-efficacy at T2 (β = 0.112, *p* = 0.004) and physical activity participation at T2 (β = 0.143, *p* < 0.001); exercise self-efficacy at T1 positively predicted Exercise Identity at T2 (β = 0.098, *p* = 0.025) and physical activity participation at T2 (β = 0.170, *p* < 0.001); and physical activity participation at T1 positively predicted Exercise Identity at T2 (β = 0.096, *p* = 0.021) and exercise self-efficacy at T2 (β = 0.172, *p* < 0.001). Although these associations were statistically significant, the effect sizes were relatively small, suggesting modest prospective relationships among the three constructs.

**FIGURE 1:**
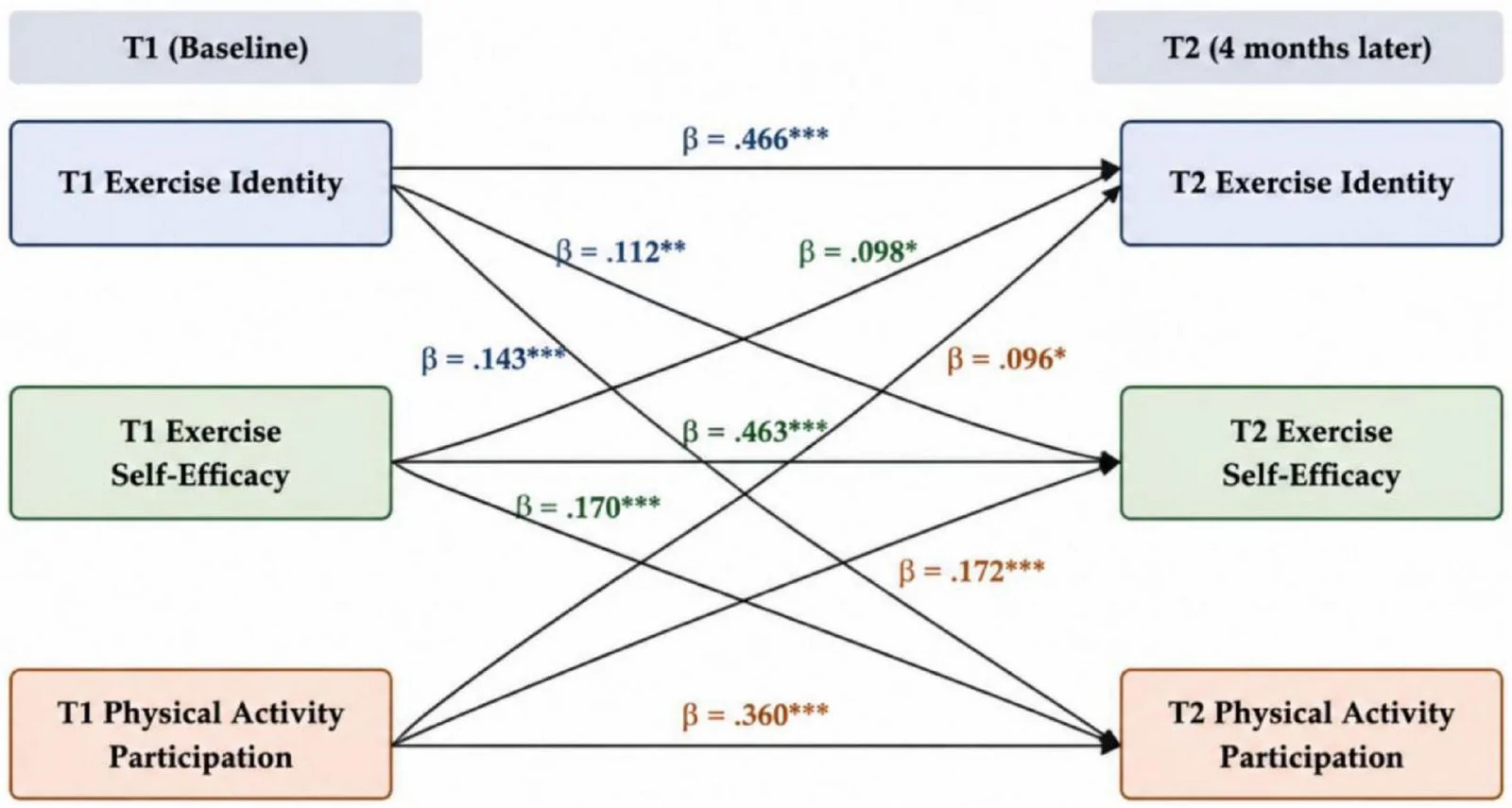
Standardized path diagram of the T1–T2 cross-lag model. The symbols *, **, and *** indicate statistical significance at *p* < 0.1, *p* < 0.05, and *p* < 0.01, respectively.

Furthermore, the model explained variance in all three T2 endogenous variables, accounting for 29.7% of the variance in Exercise Identity, 35.5% in exercise self-efficacy, and 26.2% in physical activity participation. Overall, the cross-lagged panel model results support the presence of dynamic longitudinal associations among Exercise Identity, exercise self-efficacy, and physical activity participation.

### 3.6 Testing the indirect association of exercise self-efficacy

Based on the findings from the cross-lagged panel model, an indirect effect analysis was conducted using the bootstrap method to examine whether exercise self-efficacy represents a potential explanatory pathway linking Exercise Identity and physical activity participation. In this analysis, T2 exercise self-efficacy was specified as the mediator, while baseline levels of exercise self-efficacy and physical activity participation at T1 were controlled. The bootstrap procedure was performed using 5,000 resamples to estimate the indirect effect and its 95% confidence interval. The results are presented in Table 8.

**TABLE 8:** Longitudinal mediating effect of exercise self-efficacy.

effect path	Standardized effect	*p*	Bootstrap 95% CI
T1 Exercise Identity → T2 Exercise self-efficacy (*a*)	0.112	0.006	[0.011, 0.066]
T2 Exercise self-efficacy → T2 Physical activity participation (*b*)	0.33	<0.001	
T1 Exercise Identity → T2 Physical activity participation (Direct effect, *c’*)	0.106	0.01	
Indirect effect (*a* × *b*)	0.037	–	

The Bootstrap procedure was conducted with 5,000 resamples.

The results indicated that Exercise Identity at T1 significantly and positively predicted exercise self-efficacy at T2 (a = 0.112, *p* = 0.006), and exercise self-efficacy at T2 significantly predicted physical activity participation at T2 (b = 0.330, *p* < 0.001). The direct effect of Exercise Identity at T1 on physical activity participation at T2 remained significant (c’ = 0.106, *p* = 0.010). Further analysis showed that the indirect effect of exercise self-efficacy was 0.037, with a 95% Bootstrap confidence interval (CI) of [0.011, 0.066], which excluded zero, indicating a significant indirect effect. Therefore, exercise self-efficacy partially mediated the longitudinal association between Exercise Identity and physical activity participation.

### 3.7 Summary of hypothesis testing

Table 9 summarizes the results of hypothesis testing in this study. The results indicated that all proposed hypotheses (H1–H5) were supported. Specifically, Exercise Identity, exercise self-efficacy, and physical activity participation were significantly and positively correlated across both measurement waves, and the cross-lagged panel model revealed significant longitudinal predictive associations among these constructs. Furthermore, longitudinal mediation analysis indicated that exercise self-efficacy partially mediated the longitudinal association between Exercise Identity and physical activity participation.

**TABLE 9:** Summary of hypothesis testing results.

Hypothesis	Statistical results	Conclusion
H1	Exercise Identity, exercise self-efficacy, and physical activity participation were significantly positively correlated across both measurement waves.	Supported
H2	T1 Exercise Identity positively predicted T2 physical activity participation (β = 0.143, *p* < 0.001).	Supported
H3	T1 physical activity participation positively predicted T2 Exercise Identity (β = 0.096, *p* = 0.021).	Supported
H4	T1 Exercise Identity positively predicted T2 exercise self-efficacy (β = 0.112, *p* = 0.004).	Supported
H5	Exercise self-efficacy partially mediated the longitudinal association between Exercise Identity and physical activity participation (indirect effect = 0.037, 95% Bootstrap CI [0.011, 0.066]).	Supported

Overall, findings from the two-wave longitudinal dataset indicated that Exercise Identity, exercise self-efficacy, and physical activity participation demonstrated both concurrent associations and longitudinal predictive associations after accounting for the temporal stability of each construct. Specifically, Exercise Identity at T1 predicted subsequent physical activity participation at T2 and exerted an indirect association through exercise self-efficacy; meanwhile, physical activity participation at T1 predicted subsequent Exercise Identity and exercise self-efficacy at T2. These findings provide empirical support for the proposed dynamic reciprocal model linking Exercise Identity, exercise self-efficacy, and physical activity participation.

## 4 Discussion

Guided by identity theory and social cognitive theory, this study examined the prospective reciprocal associations among college students’ Exercise Identity, exercise self-efficacy, and physical activity participation using a two-wave longitudinal design. The findings suggest that these constructs were not exclusively related in a unidirectional manner but showed reciprocal longitudinal associations across measurement occasions. Specifically, Exercise Identity was prospectively associated with later physical activity participation and showed an indirect association through exercise self-efficacy, whereas physical activity participation was associated with subsequent Exercise Identity and exercise self-efficacy. Given the two-wave design, these findings should be interpreted as prospective longitudinal associations rather than evidence of causal developmental processes. Overall, the results highlight the interconnected relationships among identity-related perceptions, competence beliefs, and physical activity participation among college students.

### 4.1 Exercise identity as an important psychological foundation for physical activity participation

This study found that, after controlling for the temporal stability of physical activity participation, Exercise Identity was positively associated with subsequent physical activity participation. This finding suggests that sustained physical activity participation among college students may be related not only to immediate motivation or enjoyment but also to the extent to which exercise is incorporated into individuals’ self-concepts.

From the perspective of Exercise Identity theory, this association may be understood through the role of identity-consistent behavior. When individuals perceive exercise as an important part of who they are, they may be more likely to engage in behaviors that are consistent with this self-perception. In addition, a stronger Exercise Identity may be associated with greater persistence when encountering common barriers to physical activity, such as academic demands, limited time availability, and changes in daily routines (Liu et al., 2026).

These findings suggest that Exercise Identity represents an important psychological factor associated with physical activity participation. Recent research has increasingly emphasized identity-based perspectives in understanding physical activity maintenance, highlighting the relevance of Exercise Identity in explaining behavioral persistence (Porter et al., 2024). Rather than demonstrating a causal mechanism, the present findings provide longitudinal evidence that Exercise Identity is prospectively associated with physical activity participation among college students.

### 4.2 Physical activity participation as a behavioral experience associated with exercise identity

Beyond the traditional perspective that psychological factors precede behavioral engagement, this study further suggests that physical activity participation may also be associated with subsequent levels of Exercise Identity. This finding indicates that Exercise Identity may not only relate to physical activity behavior but may also be associated with individuals’ later perceptions of themselves as exercisers.

From a psychological perspective, repeated participation in physical activity may provide individuals with opportunities to accumulate exercise-related experiences, improve perceived competence, and develop stronger connections with exercise-related roles. For college students, successful completion of exercise activities, positive physical experiences, and social interactions within exercise contexts may contribute to changes in how they perceive their relationship with physical activity.

This finding suggests that promoting physical activity participation may involve not only encouraging identity-related beliefs but also creating opportunities for meaningful and positive exercise experiences. When students repeatedly engage in physical activity, these experiences may be associated with stronger Exercise Identity over time. Recent research has also examined reciprocal associations between physical activity behavior and identity-related factors (Wang et al., 2026). The present findings are consistent with a reciprocal association perspective, while further research with more measurement occasions is needed to clarify developmental changes in Exercise Identity.

### 4.3 Exercise self-efficacy as a potential explanatory pathway linking Exercise identity and physical activity participation

This study found that exercise self-efficacy showed a significant indirect association in the relationship between Exercise Identity and physical activity participation. This finding suggests that Exercise Identity may be associated with physical activity participation partly through individuals’ confidence in their ability to engage in exercise behaviors.

From a psychological perspective, Exercise Identity reflects the question of “Who am I as an exerciser?”, whereas exercise self-efficacy reflects the belief of “Can I successfully engage in exercise behaviors?”. These constructs represent related but distinct psychological processes, with identity reflecting self-definition and self-efficacy reflecting perceived capability.

Individuals with a stronger Exercise Identity may place greater personal value on exercise, which may be associated with stronger confidence in maintaining physical activity behaviors. In turn, higher exercise self-efficacy may support continued engagement when individuals encounter barriers such as limited time, fatigue, or competing demands (Kong and Niu, 2026). Previous research among college students has similarly highlighted the importance of self-efficacy in understanding the relationship between psychological beliefs and physical activity participation (Yu et al., 2024; Li et al., 2024).

However, because exercise self-efficacy and physical activity participation were assessed concurrently at T2, this indirect association should not be interpreted as evidence of a fully temporally ordered causal mediation process. Instead, it represents a potential explanatory pathway that requires further examination using designs with additional measurement occasions.

### 4.4 Reciprocal longitudinal associations between psychological factors and physical activity participation

A central finding of this study is that Exercise Identity, exercise self-efficacy, and physical activity participation showed reciprocal longitudinal associations across the two measurement occasions. The cross-lagged panel model demonstrated that all six cross-lagged paths were statistically significant, suggesting that psychological factors and behavioral experiences were prospectively associated with each other over time.

These findings extend traditional approaches that primarily focus on psychological factors as predictors of physical activity behavior by suggesting that behavioral experiences may also be associated with subsequent psychological characteristics. Physical activity participation may provide experiences that are related to individuals’ confidence and identity perceptions, whereas stronger identity and competence beliefs may be associated with continued participation.

However, the magnitude of the cross-lagged coefficients was relatively small, indicating modest prospective associations among the three constructs. Therefore, these relationships should be interpreted cautiously, and physical activity participation is likely influenced by multiple psychological, social, and environmental factors.

From a practical perspective, these findings may inform approaches that consider both behavioral engagement and psychological factors when promoting physical activity among college students. Supporting positive exercise experiences, strengthening confidence in exercise abilities, and fostering exercise-related identity perceptions may represent potentially valuable components of physical activity promotion strategies.

## 5 Conclusion

This study examined the prospective reciprocal associations among Exercise Identity, exercise self-efficacy, and physical activity participation in college students using a two-wave longitudinal design. The findings showed that Exercise Identity, exercise self-efficacy, and physical activity participation were prospectively associated with one another across time, with significant reciprocal cross-lagged relationships among the three constructs. Exercise Identity was positively associated with subsequent physical activity participation, while physical activity participation was also associated with later Exercise Identity and exercise self-efficacy. In addition, exercise self-efficacy showed a significant indirect association between Exercise Identity and physical activity participation, suggesting that perceived exercise competence may represent a potential explanatory pathway linking identity-related perceptions with behavioral engagement.

Overall, these findings highlight the importance of considering Exercise Identity, exercise self-efficacy, and physical activity participation within an integrated longitudinal framework rather than as isolated or strictly unidirectional factors. However, given the two-wave observational design and the relatively small magnitude of the cross-lagged effects, the results should be interpreted as evidence of modest prospective associations rather than causal developmental processes. Future research using additional measurement occasions and more advanced longitudinal models is needed to further clarify the temporal interplay among these psychological and behavioral factors.

## 6 Limitations and future directions

Several limitations should be considered. First, this study used a two-wave longitudinal design. Although the cross-lagged panel model allowed examination of prospective associations while controlling for prior levels of the constructs, it cannot establish causal relationships or fully distinguish within-person changes from stable between-person differences. Future research using three or more measurement occasions and advanced longitudinal models, such as random-intercept cross-lagged panel models, may provide a more detailed understanding of these processes.

Second, although the observed cross-lagged associations were statistically significant, their effect sizes were relatively small. Future studies should examine additional psychological and contextual factors that may explain further variability in physical activity participation.

Third, the indirect association involving exercise self-efficacy should be interpreted cautiously because the mediator and outcome were measured at the same time point. Future studies using designs with greater temporal separation among variables are needed to clarify the potential explanatory role of exercise self-efficacy.

Finally, the sample consisted exclusively of college students, which may limit the generalizability of the findings to other populations. Participants also varied in gender, age, year of study, and academic field, and these characteristics may influence exercise opportunities, academic demands, and psychological responses to physical activity. Their potential moderating effects were not examined in the present study. Future research should assess whether the observed associations differ across demographic and academic subgroups.

## Data Availability

The raw data supporting the conclusions of this article will be made available by the authors, without undue reservation.
